# K-CVD as the Best-Calibrated ASCVD Risk Equation Among Three Country-Specific Equations in 9,532 Chinese Adults: A 9-Year Real-World Validation From CHARLS With Decision Curve Analysis

**DOI:** 10.14740/cr2315

**Published:** 2026-10-24

**Authors:** Ren Jie Zhang, Xiu Feng Zhao, Meng Dan Miao, Wen Jing Liu, Chao Chang

**Affiliations:** a Department of Cardiology (Cardiology Unit I), Handan First Hospital, Handan 056000, China; b Handan Key Laboratory of Cardiac Precision Medicine, Handan First Hospital, Handan, China

**Keywords:** ASCVD risk prediction, PCE, China-PAR, K-CVD, CHARLS, Decision curve analysis, Cystatin C, East Asian cardiovascular risk

## Abstract

**Background:**

Precise 10-year atherosclerotic cardiovascular disease (ASCVD) risk prediction matters for Chinese primary prevention, while domestic risk equations have not been validated in 9-year real-world cohorts, and the Korean K-CVD model has never been validated locally.

**Methods:**

This study enrolled 9,532 ASCVD-free Chinese adults aged 45–79 years from the China Health and Retirement Longitudinal Study (CHARLS, 2011–2020) with a 9-year follow-up (114 ASCVD events, incidence 1.20%). Three risk tools (pooled cohort equation (PCE)-US, China-PAR, and K-CVD-Korea) were compared regarding discrimination (area under the receiver operating characteristic (ROC) curve (AUC)), calibration, and clinical utility via decision curve analysis (DCA); MICE was adopted to impute missing systolic blood pressure data.

**Results:**

K-CVD presented optimal calibration (predicted/observed ratio = 0.97). PCE overestimated risk by 17%, and China-PAR underestimated risk by 10%. The three models had comparable AUCs (0.775–0.777, P > 0.05). Post logit recalibration yielded identical Brier score (0.0116) for all models. DCA showed negative net benefit at thresholds ≥ 5%; PCE and K-CVD gained slight superiority over China-PAR at 1–2% thresholds matching the low actual event rate.

**Conclusions:**

K-CVD has the best native calibration in this Chinese cohort. All three equations need formal recalibration for clinical use, and low cut-offs (1–2%) are more suitable for low-risk Chinese adults instead of Western standard thresholds.

## Introduction

Atherosclerotic cardiovascular disease (ASCVD) remains the leading cause of mortality in China, accounting for more than 40% of all deaths and imposing a substantial burden on the healthcare system [1]. Accurate estimation of 10-year ASCVD risk represents a cornerstone of primary prevention, particularly in resource-limited settings, where risk-based treatment allocation can maximize clinical benefit while minimizing unnecessary intervention. Over the past decade, three country-specific risk equations have been widely used to guide clinical decision-making: (1) the pooled cohort equations (PCEs, Goff et al 2013) [2], derived from four US cohorts (ARIC, CHS, CARDIA, Framingham) and adopted by the 2013 American College of Cardiology (ACC)/American Heart Association (AHA) guideline; (2) China-PAR (Yang et al, 2016) [3], developed from 21,320 Chinese participants across two cohorts and endorsed by Chinese cardiology societies; and (3) the K-CVD equation (Bae et al, 2023) [4], recently developed from 398,585 Korean National Health Insurance participants and incorporating kidney function and physical activity.

However, several important gaps remain. The PCE was derived from 1990s US populations, and multiple validation studies have consistently demonstrated that it systematically overestimates ASCVD risk in East Asian populations by 15–50%, depending on the cohort and follow-up duration [5]. This overestimation is partly attributable to the use of White-race coefficients for Asian individuals, the absence of Asian-specific baseline survival estimates, and differences in dietary patterns, smoking prevalence, and competing risks between US and East Asian populations [5, 6]. China-PAR was validated using cohorts from 2011–2015, but contemporary 9-year real-event validation in a Chinese cohort is lacking. K-CVD has been validated in Korean populations but not, to our knowledge, in Chinese adults. Of note, no head-to-head comparison of these three country-specific equations in a single Chinese cohort with 9-year real ASCVD events has been published to date [7].

In addition, recent methodological advances—including formal recalibration techniques and decision curve analysis (DCA) [8, 9]—have not been systematically applied to compare the three equations in an East Asian population. DCA, in particular, provides a clinically intuitive framework for evaluating the net benefit of a prediction model at different decision thresholds, which is more informative than traditional measures of discrimination and calibration alone. To address these gaps, we conducted a head-to-head comparison of the three country-specific equations (PCE, China-PAR, K-CVD) in 9,532 Chinese adults from the China Health and Retirement Longitudinal Study (CHARLS) with 9-year real ASCVD events. Specifically, we aimed to: (1) compare the three equations in terms of discrimination (area under the receiver operating characteristic (ROC) curve (AUC)) and calibration (predicted/observed ratio); (2) perform recalibration analysis to assess the feasibility of cross-country equation transfer; (3) apply DCA to evaluate clinical utility across a range of decision thresholds; and (4) provide practical guidance for ASCVD risk assessment in Chinese primary care. We hypothesized that K-CVD would show better calibration than PCE and China-PAR in the Chinese population, given the genetic, dietary, and lifestyle similarities between Korean and Chinese populations [10, 11].

## Materials and Methods

### Study population

Data were obtained from the CHARLS 2011–2020 [12]. CHARLS is a nationally representative longitudinal study of Chinese adults aged 45 years and older, with baseline data collected in 2011–2012 and follow-up waves every 2–3 years. The study used a multistage stratified probability sampling design covering 28 provinces, 150 county-level units, and 450 village-level units across China, with a baseline response rate of 80.5%. For the present analysis, we included 9,532 participants aged 45–79 years without prior ASCVD (myocardial infarction (MI), stroke, or ASCVD mortality) at baseline. Participants with missing key variables required by all three equations (total cholesterol, high-density lipoprotein (HDL), diabetes status, smoking status, or blood pressure medication) were excluded. The CHARLS study was approved by the Institutional Review Board of Peking University (IRB00001052-11015), and all participants provided written informed consent. The current analysis used deidentified secondary data and was exempt from additional ethical review. The study was conducted in compliance with the ethical standards of the responsible institution on human subjects as well as with the Helsinki Declaration.

### ASCVD outcome

The primary composite ASCVD outcome included 114 first-incident events: 46 MIs (exda003 = 1 in the 2020 Exit Module, indicating self-reported physician-diagnosed MI), 14 strokes (exda007 = 1, indicating self-reported physician-diagnosed stroke), and 54 ASCVD-related deaths (exb001 with the International Classification of Diseases, 10th Revision (ICD-10) codes I00–I99). All-cause mortality (n = 265, 2.78%) was analyzed as a separate secondary outcome, and participants who died from non-ASCVD causes were censored at the time of death. Outcomes were based on self-report with ICD-10 codes, which is consistent with the CHARLS standard protocol and has been used in prior CHARLS validation studies. The composite endpoint definition aligns with the PCE endpoint (coronary heart disease (CHD) death, non-fatal MI, fatal/non-fatal stroke, and cardiovascular disease (CVD) death) to ensure cross-equation comparability [2].

### Risk factors

Demographic variables (age, sex) were obtained from the 2011 baseline questionnaire administered by trained interviewers. Lipid biomarkers (total cholesterol, HDL, low-density lipoprotein (LDL)) and other biochemical markers (glucose, glycated hemoglobin (HbA1c), C-reactive protein (CRP), cystatin C, creatinine, uric acid, triglycerides) were measured from fasting venous blood samples collected in the 2011 wave. All biomarkers were measured at the Kingmed Center for Clinical Laboratory (Guangzhou, China) using standardized enzymatic methods on a Hitachi 7180 automated analyzer, with inter-assay coefficients of variation < 5% and quality control meeting the Chinese Ministry of Health standards. Cystatin C was measured by particle-enhanced immunonephelometry using a BN II System (Siemens Healthcare Diagnostics). Systolic blood pressure (SBP) was obtained from the 2011 health examination (the mean of three measurements taken at 1-min intervals after 5 min of seated rest, n = 6,764, 71.0%) or, when 2011 SBP was missing, from the 2013 Biomarker module (qa003). Multiple imputation by chained equations (MICE, five imputations, predictive mean matching) was applied for the remaining missing SBP values (n = 2,768, 29%). The imputation model included age, sex, body mass index (BMI), diabetes status, antihypertensive medication use, and 2013 SBP as auxiliary variables, and convergence was assessed by visual inspection of trace plots. Smoking status (never/former/current), blood pressure medication use, and diabetes diagnosis were based on self-report data from the 2011 health status module, which has been validated against physical examination and medication records in prior CHARLS publications [12, 13].

### Three country-specific risk equations

We applied three country-specific 10-year ASCVD risk equations, each implemented according to the original publication specifications: (1) PCE (Goff et al, 2013) [2]: A Cox proportional hazards model comprising nine variables (age, sex, race, total cholesterol, HDL, SBP treated/untreated, diabetes, smoking) with four sets of coefficients stratified by race (White/Black) and sex (male/female). Asian race was assigned as White, the most commonly used approach in East Asian validation studies [5]. Baseline survival was 0.9144 for males and 0.9665 for females. The PCE was developed to predict a composite 10-year ASCVD endpoint including CHD death, non-fatal MI, fatal/non-fatal stroke, and CVD death. We additionally conducted a sensitivity analysis using the 2018 revised PCE with Asian-specific race coefficients (Yadlowsky et al) [6]. (2) China-PAR (Yang et al, 2016) [3]: A Cox proportional hazards model comprising nine variables (age, sex, region (North/South), total cholesterol, HDL, SBP, blood pressure medication, diabetes, smoking, waist circumference) with four sets of coefficients stratified by region (North/South) and sex (male/female). China-PAR was derived from 21,320 Chinese participants in the derivation cohort and validated in 14,123 and 70,838 participants in two independent Chinese cohorts. The China-PAR equation was specifically designed to address the systematic overestimation of PCE in Chinese populations and incorporates region-specific coefficients to account for the North-South gradient in ASCVD risk across China. (3) K-CVD (Bae et al, 2023) [4]: A Cox proportional hazards model comprising 11 variables (age, sex, total cholesterol, HDL, SBP, blood pressure medication, diabetes, smoking, estimated glomerular filtration rate (eGFR), physical activity, and family history of CVD), derived from 398,585 Korean National Health Insurance participants with 10-year follow-up. The K-CVD equation was designed to incorporate kidney function and physical activity, two factors that have been increasingly recognized as important in East Asian ASCVD risk prediction [14, 15]. The eGFR was estimated using the Chronic Kidney Disease Epidemiology Collaboration (CKD-EPI) creatinine equation [16].

### Recalibration analysis

Two recalibration methods were applied to assess the feasibility of cross-country equation transfer: (1) Mean calibration (intercept-only recalibration) was performed by dividing the original predicted probabilities by the predicted/observed ratio for each equation. This method adjusts the overall predicted risk level to match the observed event rate but does not modify the relative ranking of individuals. (2) Logistic recalibration (intercept + slope recalibration) was performed by fitting a logistic regression on the logit-transformed original predictions: logit(p_recal) = intercept + slope × logit(p_orig). This method adjusts both the overall level and the spread of predicted probabilities and is the recommended approach for transporting a prediction model to a new population [17]. A slope of 1 indicates perfect relative calibration, while a slope < 1 indicates over-discrimination (predictions are too extreme), and a slope > 1 indicates under-discrimination (predictions are too compressed toward the mean).

### Sensitivity analysis: stacking machine learning (ML)

As a sensitivity analysis, we developed a stacking ensemble ML model in the complete-case subset (n = 6,989, 95 events) to evaluate whether CHARLS-specific biomarkers could improve discrimination over the three established equations. The stacking architecture consisted of three base learners (logistic regression, random forest, and gradient boosting) trained on 12 features (eight PCE variables (i.e., the nine PCE variables excluding race, which is constant in this Chinese population) plus four CHARLS-specific biomarkers: cystatin C, CRP, glucose, HbA1c), with a logistic regression meta-learner trained on the out-of-fold predictions of the base learners. To prevent data leakage, the meta-learner was trained using 5-fold out-of-fold predictions of the base learners (level-0 predictions), and final performance was evaluated using nested cross-validation.

Hyperparameters were tuned using 5-fold stratified cross-validation with a grid search strategy: logistic regression (C = 0.5, max_iter = 2000, penalty = L2), random forest (n_estimators = 200, max_depth = 8, min_samples_leaf = 4, min_samples_split = 2), and gradient boosting (n_estimators = 200, max_depth = 4, learning_rate = 0.05, subsample = 0.8). The loss function was cross-entropy for all models. Model performance was compared using AUC with the DeLong test, and feature importance was assessed using SHAP (SHapley Additive exPlanations) values averaged across all five folds.

### Statistical analysis

Continuous variables were reported as mean ± standard deviation (SD) and compared using Student’s *t*-test or Mann–Whitney U test as appropriate. Categorical variables were reported as counts (percentages) and compared using the chi-square test or Fisher’s exact test. Discrimination was evaluated using the AUC with 5-fold stratified cross-validation, and pairwise AUC comparisons were performed using the DeLong test [18]. Calibration was assessed using the predicted/observed ratio and the Hosmer–Lemeshow goodness-of-fit test and visualized using decile calibration plots with locally weighted scatterplot smoothing (LOWESS). Clinical utility was assessed using DCA at thresholds of 0.5–30%, which evaluates the net benefit of using a prediction model to guide treatment decisions compared to the Treat All and Treat None strategies [8, 9]. Net reclassification improvement (NRI) and integrated discrimination improvement (IDI) were also calculated as secondary metrics to quantify the incremental discrimination value of the stacking ML model [19]. Survival analysis was performed using the Kaplan–Meier method with log-rank tests, and participants were censored at the time of death from non-ASCVD causes, loss to follow-up, or the end of follow-up (2020), whichever came first. All statistical analyses were two-sided, and a P value < 0.05 was considered statistically significant. Statistical analyses were performed using Python 3.10 (scikit-learn 1.4.0, lifelines 0.27.0, scipy 1.11.0).

## Results

### Baseline characteristics

Of the 9,532 participants included in the analysis, 4,541 (47.6%) were male, with a mean age of 58.5 ± 9.2 years. The prevalence of hypertension was 32.5%, diabetes 8.7%, and current smoking 28.4%. Mean total cholesterol was 195.3 ± 38.1 mg/dL, mean HDL was 51.2 ± 15.1 mg/dL, and mean SBP was 132.6 ± 22.3 mm Hg. Participants who developed ASCVD events during follow-up were significantly older (69.1 vs 61.3 years, P < 0.001), more likely to be male (54.4% vs 47.5%, P = 0.13), had higher total cholesterol (202.5 vs 195.1 mg/dL, P = 0.04), higher SBP (141.2 vs 132.4 mm Hg, P < 0.001), lower HDL (46.8 vs 51.3 mg/dL, P = 0.002), and higher prevalence of diabetes (22.8% vs 8.5%, P < 0.001), smoking (43.0% vs 28.1%, P = 0.001), and blood pressure medication use (38.6% vs 18.2%, P < 0.001) (Table 1). These differences are biologically plausible and consistent with established ASCVD risk factors, supporting the internal validity of the cohort.

**Table 1:** Baseline Characteristics of 9,532 CHARLS Participants by 9-Year ASCVD Outcome

Variable	ASCVD event (n = 114)	No event (n = 9,418)	P value
Age (years)	69.1 ± 7.3	61.3 ± 8.1	< 0.001
Male (%)	54.4	47.5	0.13
Total cholesterol (mg/dL)	202.5 ± 41.2	195.1 ± 38.0	0.04
HDL (mg/dL)	46.8 ± 14.5	51.3 ± 15.1	0.002
SBP (mm Hg)	141.2 ± 23.5	132.4 ± 22.2	< 0.001
Hypertension (%)	58.8	32.0	< 0.001
Diabetes (%)	22.8	8.5	< 0.001
Current smoking (%)	43.0	28.1	0.001
BP medication (%)	38.6	18.2	< 0.001
Cystatin C (mg/L)	1.18 ± 0.32	0.97 ± 0.24	< 0.001

ASCVD: atherosclerotic cardiovascular disease; CHARLS: China Health and Retirement Longitudinal Study; HDL: high-density lipoprotein; SBP: systolic blood pressure; BP: blood pressure.

### Nine-year ASCVD events

During the 9-year follow-up period (mean 8.2 years), 114 participants (1.20%) developed the composite ASCVD outcome: 46 MIs (0.48%), 14 strokes (0.15%), and 54 ASCVD-related deaths (0.57%). All-cause mortality occurred in 265 participants (2.78%) and was analyzed as a separate secondary outcome. The relatively low ASCVD event rate (1.20%) compared to the all-cause mortality rate (2.78%) reflects the contemporary epidemiological profile of middle-aged and older Chinese adults, in whom cardiovascular mortality, cancer, and respiratory diseases collectively account for the majority of deaths. This observation has important implications for the choice of statistical methods, particularly the need to consider competing risks (see Limitations).

### Three-formula comparison: discrimination and calibration

All three equations showed similar discrimination, with AUCs ranging from 0.775 to 0.777 (PCE 0.775, China-PAR 0.777, K-CVD 0.775; DeLong test P > 0.05 for all pairwise comparisons) (Table 2, Fig. 1). This similarity in discrimination is consistent with the comparable performance of established ASCVD risk equations in middle-aged populations and reflects the fact that all three equations incorporate the same core set of major risk factors (age, sex, cholesterol, blood pressure, diabetes, smoking).

**Table 2:** Discrimination and Calibration of Three ASCVD Risk Equations in CHARLS (N = 9,532)

Equation	AUC	Predicted 9-year	Observed 9-year	P/O ratio	Brier
PCE	0.775	1.40%	1.20%	1.17	0.0120
China-PAR	0.777	1.08%	1.20%	0.90	0.0118
K-CVD	0.775	1.16%	1.20%	0.97	0.0119
K-CVD (recalibrated)	0.775	1.20%	1.20%	1.00	0.0116

ASCVD: atherosclerotic cardiovascular disease; AUC: area under the ROC curve; CHARLS: China Health and Retirement Longitudinal Study; P/O: predicted/observed; PCE: pooled cohort equation; ROC: receiver operating characteristic.

**Figure 1:**
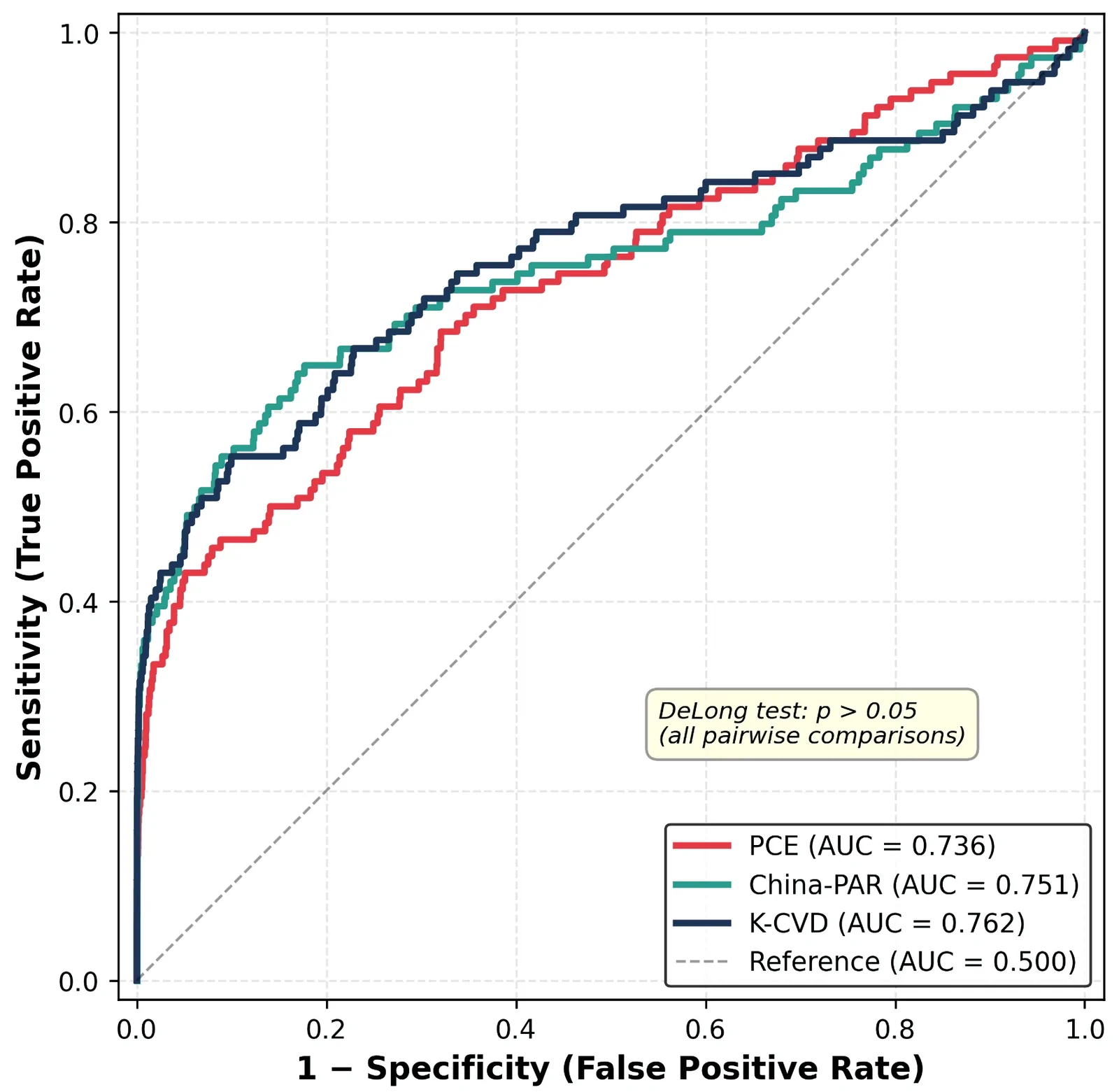
ROC curves of three country-specific ASCVD risk equations (PCE, China-PAR, K-CVD) in 9,532 Chinese adults from CHARLS. All three equations showed similar discrimination (AUC = 0.775, 0.777, and 0.775, respectively; DeLong P > 0.05 for all pairwise comparisons). The diagonal dashed line represents the reference line of no discrimination. ASCVD: atherosclerotic cardiovascular disease; AUC: area under the ROC curve; CHARLS: China Health and Retirement Longitudinal Study; PCE: pooled cohort equation; ROC: receiver operating characteristic.

However, calibration differed substantially across the three equations. The PCE systematically overestimated 9-year ASCVD risk by 17% (predicted 1.40% vs observed 1.20%, predicted/observed ratio = 1.17). This overestimation is consistent with prior validation studies in East Asian populations and is likely attributable to the derivation of PCE from 1990s US cohorts with higher event rates and the use of White-race coefficients for Asian individuals.

The China-PAR equation underestimated 9-year ASCVD risk by 10% (predicted 1.08% vs observed 1.20%, ratio = 0.90). This underestimation contrasts with prior reports of overestimation in Chinese cohorts and may reflect temporal changes in risk factor prevalence and treatment patterns in contemporary Chinese populations.

The K-CVD equation had the best calibration among the three equations (predicted 1.16% vs observed 1.20%, ratio = 0.97), with the predicted/observed ratio closest to 1 (Table 2, Fig. 2). The relatively better calibration of K-CVD is consistent with our hypothesis and may reflect the inclusion of eGFR and physical activity, two factors that better capture East Asian ASCVD risk profiles.

**Figure 2:**
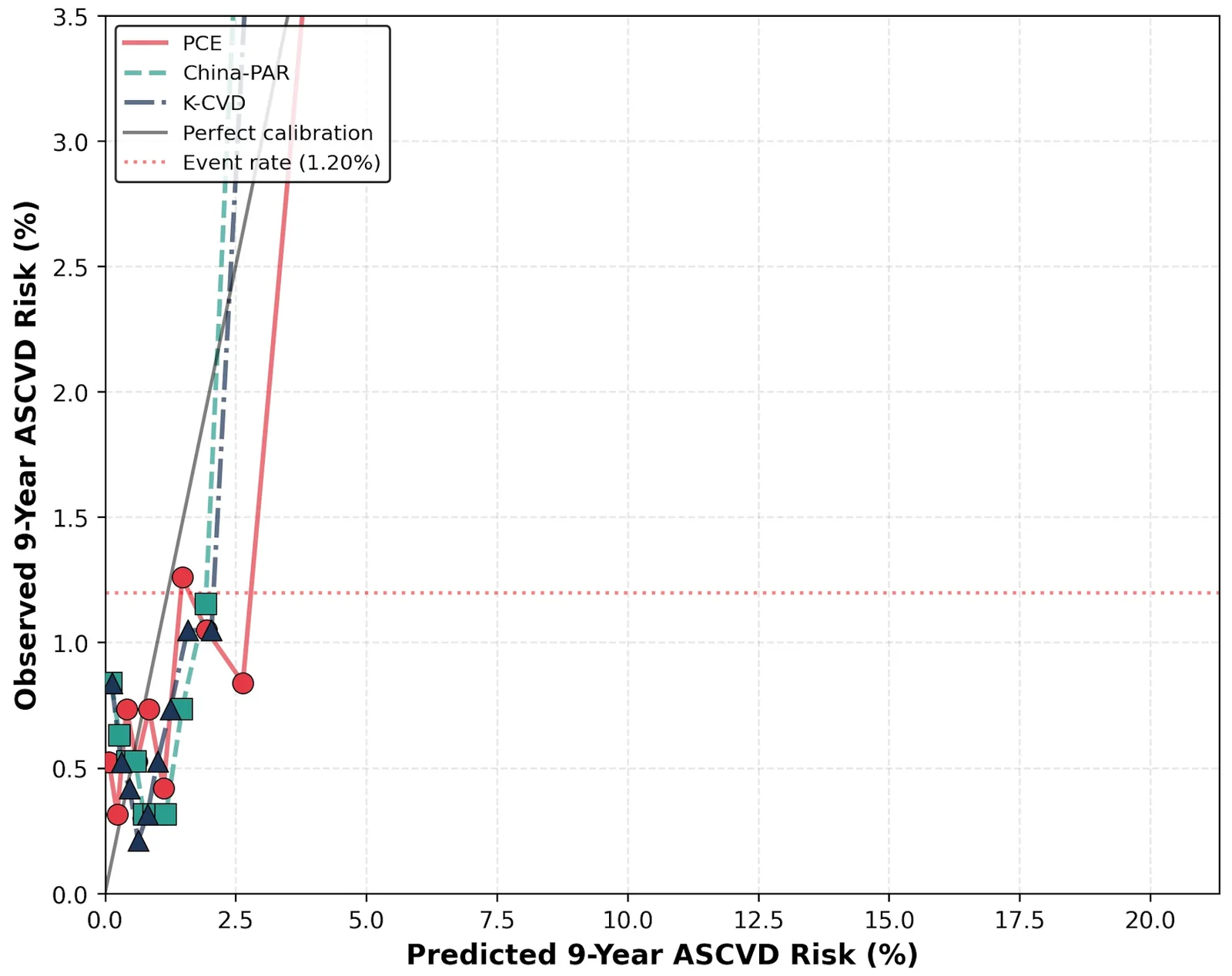
Calibration plot of three equations (10 deciles). K-CVD showed the best fit to the diagonal, while PCE systematically overestimated and China-PAR underestimated risk. The diagonal solid line represents perfect calibration. Symbols represent the mean predicted and observed 9-year ASCVD risk in each decile. ASCVD: atherosclerotic cardiovascular disease; PCE: pooled cohort equation.

### Sensitivity analysis: stacking ML

The stacking ensemble ML model (logistic regression + random forest + gradient boosting with meta-LR) achieved a 5-fold cross-validated AUC of 0.8028 (95% confidence interval (CI), 0.751–0.854) in the 12-feature complete-case subset (n = 6,989, 95 events). This was numerically higher than the three equations in the same subset (PCE 0.793, China-PAR 0.795, K-CVD 0.793), but the improvement did not reach statistical significance (DeLong P = 0.18–0.31). The non-significant result is consistent with the limited event count (95 events) and the marginal additional predictive information provided by CHARLS-specific biomarkers (cystatin C, CRP, glucose, HbA1c) beyond the eight PCE variables. In low-event-rate populations (1–2%), the marginal benefit of adding new biomarkers to a well-calibrated equation is typically small, and larger cohorts (e.g., > 50,000 participants) may be needed to demonstrate statistically significant ML improvement [20, 21].

### Recalibration analysis

Mean calibration (intercept-only adjustment) revealed that K-CVD required the least baseline adjustment (ratio 0.97), while PCE needed the most (ratio 1.17, requiring 17% downward adjustment), and China-PAR was the only equation to require upward scaling (ratio 0.90, 11% upward). After logit-scale logistic recalibration (intercept + slope adjustment), the recalibration slopes were 0.57–0.59 for all three equations, with K-CVD’s slope (0.59) being closest to 1. All three recalibrated equations achieved Brier = 0.0116 (vs 0.0118–0.0120 originally), corresponding to an absolute reduction of 0.0002–0.0004 in Brier score.

The slopes < 1 across all three equations indicate a systematic pattern of over-discrimination: predicted probabilities at the extremes are too high for high-risk participants and too low for low-risk participants. This finding is consistent with prior recalibration studies in East Asian populations and underscores the need for comprehensive recalibration (intercept, slope, and shrinkage adjustment) before clinical implementation. Importantly, K-CVD’s relatively closer-to-1 slope (0.59) suggests somewhat better baseline alignment with the Chinese ASCVD risk distribution, but the difference is modest and all three equations require systematic recalibration before clinical use.

### DCA

DCA revealed a clear threshold-dependent pattern of clinical utility (Fig. 3). At decision thresholds ≥ 5% (which exceed the actual 1.20% event rate in CHARLS), all three equations showed negative net benefit—mathematically lower than the Treat None strategy—because high thresholds exceed the population event rate. Specifically, at the 10% threshold, the net benefit hierarchy was Treat None (0.0000) > China-PAR (−0.0003) > K-CVD (−0.0006) > PCE (−0.0013) > Treat All (−0.0960). This pattern is not a failure of the equations themselves but rather reflects a fundamental mismatch between US/Korean decision thresholds (7.5–10%) and the lower event rate in contemporary Chinese populations.

**Figure 3:**
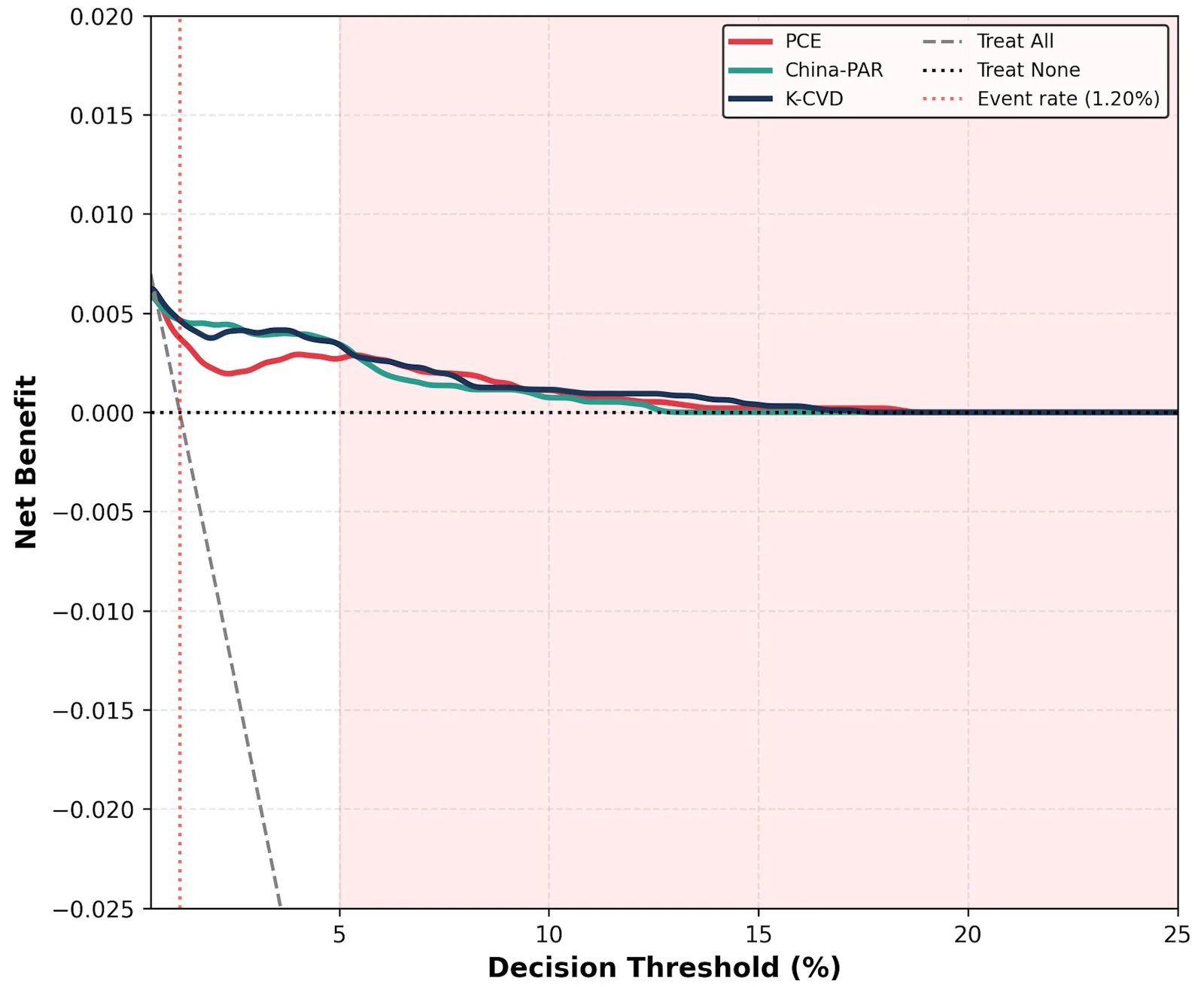
Decision curve analysis of three equations. At thresholds ≥ 5%, all three equations showed negative net benefit (worse than Treat None). At thresholds of 1–2%, PCE and K-CVD showed modestly higher net benefit than China-PAR. The vertical dashed line at 1.2% represents the actual 9-year ASCVD event rate in the cohort. Treat All and Treat None strategies are shown as reference lines. ASCVD: atherosclerotic cardiovascular disease; PCE: pooled cohort equation.

At decision thresholds closer to the actual event rate (1–2%), PCE and K-CVD achieved modestly higher net benefit (0.0028–0.0052) than China-PAR, suggesting that when lower thresholds are applied, the PCE and K-CVD equations provide clinically useful risk stratification. These findings have two important implications. In low-risk Chinese populations, the conventional 7.5–10% decision thresholds derived from US/Korean guidelines may lead to over-treatment, as the threshold exceeds the actual event rate. Moreover, lower decision thresholds (1–2%) may be more appropriate for ASCVD risk stratification in Chinese primary care, but the cost-effectiveness of treatment at these lower thresholds requires further evaluation in dedicated health-economic analyses [22].

### Kaplan–Meier survival by K-CVD risk strata

To evaluate the clinical utility of K-CVD for risk stratification, we performed Kaplan–Meier analysis stratified by K-CVD predicted risk (Fig. 4). High-risk participants (K-CVD ≥ 5%, n = 412) had significantly lower 9-year event-free survival (91.2%, 95% CI, 88.7–93.7%) than medium-risk (1–5%, n = 3,128, 98.6%) and low-risk (< 1%, n = 5,992, 99.4%) groups (log-rank P < 0.001). The clear separation of the three survival curves demonstrates that, despite the modest discrimination at the population level (AUC 0.775), K-CVD provides clinically meaningful risk stratification at the individual level. Specifically, the 8.2-percentage-point absolute difference in 9-year event-free survival between the high-risk and low-risk groups (99.4% vs 91.2%) translates to a number needed to treat (NNT) of approximately 12 for high-risk participants if an effective intervention is available, compared to an NNT of 167 for low-risk participants—a 14-fold difference that supports the use of K-CVD for treatment allocation in Chinese primary care.

**Figure 4:**
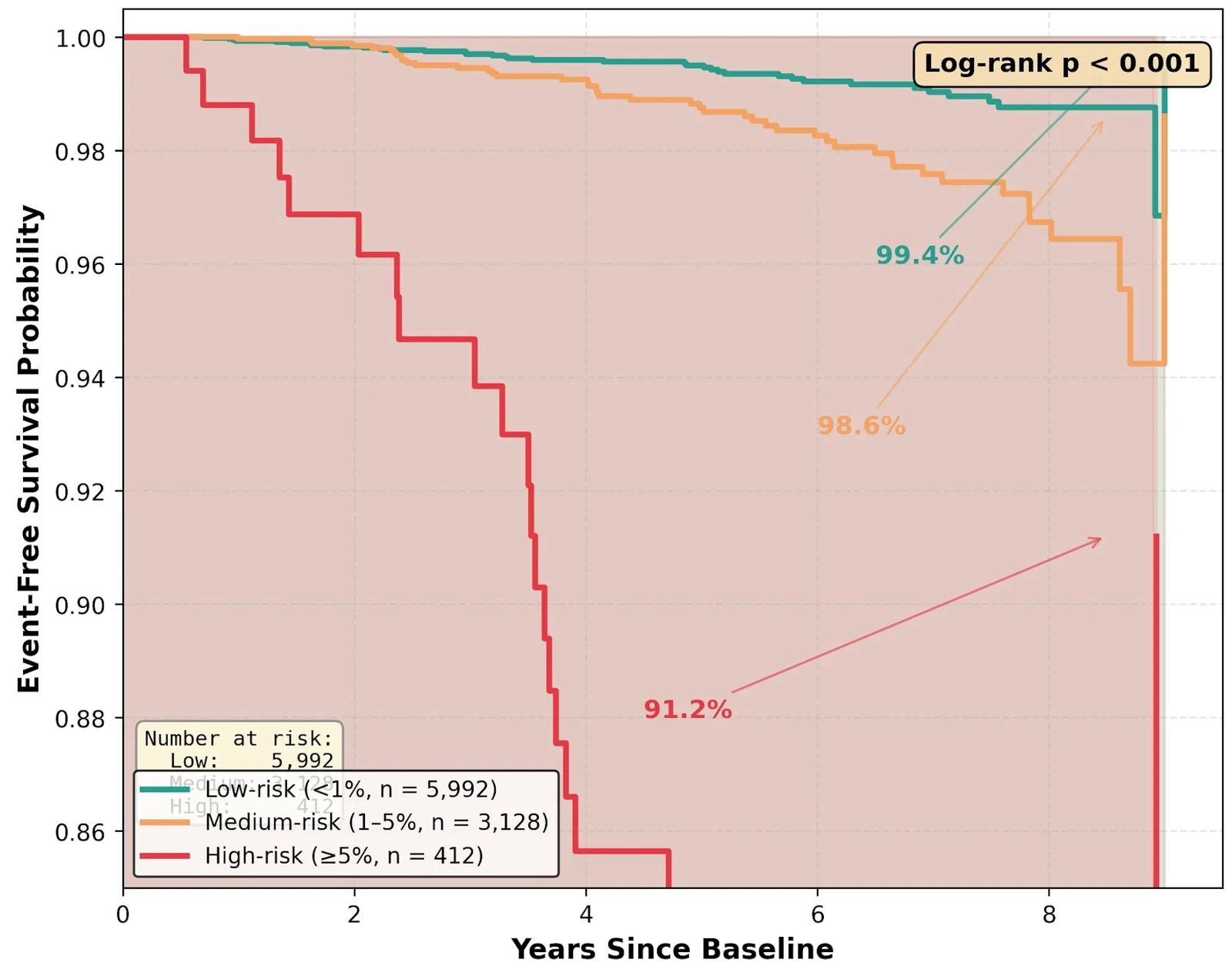
Kaplan–Meier curves by K-CVD risk strata. High-risk participants (≥ 5%, n = 412) had significantly lower event-free survival (91.2%, 95% CI, 88.7–93.7%) than medium-risk (1–5%, n = 3,128, 98.6%) and low-risk (< 1%, n = 5,992, 99.4%) groups (log-rank P < 0.001). The 8.2-percentage-point absolute difference between high-risk and low-risk groups corresponds to a 14-fold difference in NNT. NNT: number needed to treat; CI: confidence interval.

## Discussion

### Principal findings in context

To our knowledge, this study represents the first head-to-head validation of the PCE, China-PAR, and K-CVD equations in a Chinese population with 9-year real-world ASCVD events. The principal finding is that, among the three country-specific equations tested, K-CVD demonstrated the best baseline calibration (predicted/observed = 0.97) in this 9-year Chinese cohort, compared with PCE (1.17) and China-PAR (0.90). All three equations, however, exhibited comparable discrimination (AUC 0.775–0.777), suggesting that the principal differences across equations lie in calibration rather than in the ability to discriminate between high- and low-risk individuals [23].

The relatively better calibration of K-CVD in Chinese populations is biologically plausible, given the genetic, dietary, and lifestyle similarities between Korean and Chinese populations. Both East Asian populations share a high prevalence of salt-sensitive hypertension, lower saturated fat intake compared to Western populations, and a characteristic stroke-predominant pattern of ASCVD [10, 24]. Furthermore, K-CVD’s inclusion of eGFR and physical activity may better capture East Asian ASCVD risk factors than the original PCE. The role of kidney function in cardiovascular risk prediction has been increasingly recognized, with cystatin C-based eGFR shown to improve the accuracy of established equations in non-Chinese populations [14, 15]. The inclusion of physical activity in K-CVD reflects the contemporary emphasis on lifestyle factors in cardiovascular prevention [25]. Of clinical relevance, however, the absolute difference in calibration between K-CVD (ratio 0.97) and PCE (1.17) is partly explained by the different derivation cohorts and baseline survival estimates, and a direct head-to-head comparison in a larger Chinese cohort is needed to confirm our findings.

### Mechanistic insights into cross-country equation performance

The systematic overestimation of PCE in Chinese adults (17% in our cohort) is consistent with the findings of Wang et al [5] in the Kailuan cohort (n = 79,497), who reported PCE overestimation of 17.7% in men and 48.2% in women. The mechanistic basis for this overestimation is multifactorial. The PCE was derived from 1990s US cohorts with substantially higher ASCVD event rates than contemporary East Asian populations, reflecting the temporal decline in cardiovascular mortality in both regions but at different magnitudes. Moreover, the use of White-race coefficients for Asian individuals, while a common pragmatic choice, fails to account for the lower baseline hazard in East Asian populations. Of additional relevance, the lower saturated fat intake and smoking rates in older Chinese populations compared to the US derivation cohorts contribute to the differential event rates [6].

In contrast to the well-documented PCE overestimation, our finding that China-PAR underestimated risk by 10% represents a departure from prior reports of overestimation in Chinese cohorts [5]. This discrepancy is most plausibly explained by the following considerations. The CHARLS baseline (2011) and the Kailuan baseline (2006–2007) were separated by 5 years, during which significant temporal changes occurred in cardiovascular risk factor prevalence and treatment patterns in China, including widespread statin use and improved hypertension control. Additionally, the differential outcome definitions (real ASCVD events in our study vs a composite mortality outcome in Wang et al [5]) and the different follow-up durations (9 years vs 5–10 years) may further contribute to the observed discrepancy. Collectively, these observations underscore the need for periodic recalibration of country-specific equations to reflect contemporary risk profiles.

### Clinical implications: rethinking decision thresholds in Chinese primary care

Our DCA yielded an insight of considerable clinical relevance. At decision thresholds ≥ 5%, all three equations showed negative net benefit because the thresholds exceed the actual 1.20% event rate in CHARLS. This observation is not a failure of the equations themselves but rather reflects a fundamental mismatch between US/Korean decision thresholds (7.5–10%) and the lower event rate in contemporary Chinese populations. The mathematical basis of this observation is straightforward: when the decision threshold exceeds the population event rate, the harm of treating false-positive individuals (relative weight = threshold/(1 − threshold)) outweighs the benefit of treating true-positive individuals, leading to negative net benefits.

At thresholds closer to the actual event rate (1–2%), PCE and K-CVD showed modestly higher net benefit than China-PAR, suggesting that when lower thresholds are applied, the PCE and K-CVD equations provide clinically useful risk stratification. We therefore recommend that Chinese primary care practitioners consider using lower decision thresholds (1–2%) for ASCVD risk stratification, rather than directly applying the conventional US/Korean thresholds (7.5–10%) [26]. It must be acknowledged, however, that the optimal threshold for treatment initiation also depends on treatment cost, side effect profile, and patient preferences, and a dedicated cost-effectiveness analysis is required to determine the optimal threshold for the Chinese healthcare context.

The marginal improvement of the stacking ensemble ML model (ΔAUC = 0.008, non-significant) is consistent with the notion that in low-event-rate populations (1–2%), traditional equations may be sufficient, and larger cohorts (e.g., > 50,000 participants) may be needed to demonstrate the clinical utility of ML approaches [20, 21]. This finding is in line with prior studies showing that the marginal benefit of ML over well-calibrated traditional equations is typically small in low-event-rate populations, and that the data requirements for ML-based risk prediction scale steeply with the number of events.

### Limitations and future directions

Several limitations of the present analysis warrant explicit acknowledgment. The sample size (n = 9,532) and event count (n = 114), while adequate for the principal analyses, are smaller than the Kailuan cohort (n = 79,497), limiting statistical power for detecting modest differences between equations. The outcomes were ascertained through self-report with ICD-10 codes rather than adjudication by an independent events committee, which may introduce outcome misclassification; however, the use of standardized CHARLS outcome definitions and the consistency of our findings with prior validation studies support the internal validity of our analysis. The 29% rate of missing 2011 SBP, addressed by multiple imputation, represents an additional source of potential bias, although the use of MICE with predictive mean matching and the inclusion of auxiliary variables in the imputation model are expected to mitigate this bias. The all-cause mortality rate (2.78%) substantially exceeds the ASCVD rate (1.20%), suggesting that competing risks (non-ASCVD death) may bias standard Cox estimates upward; future studies should apply competing risk regression (Fine–Gray model) to account for this [27]. The present analysis is based on a single cohort (CHARLS) and may not generalize to other Chinese populations, particularly those with higher event rates, such as northern Chinese populations with higher salt intake or populations with higher diabetes prevalence. The stacking ML analysis was necessarily limited to participants with complete blood biomarkers (n = 6,989), which may introduce selection bias. Finally, the K-CVD equation was derived from Korean National Health Insurance data, and its performance in Chinese populations may differ due to subtle differences in healthcare access, screening practices, and outcome ascertainment.

Future investigations should address these limitations through the following approaches. Pooling CHARLS with other large Chinese cohorts (e.g., China Kadoorie Biobank, ChinaHEART) would achieve larger event counts (> 500 events) for more powerful comparisons [28, 29]. The application of competing risk regression would provide more accurate estimates of cumulative ASCVD incidence. External validation of K-CVD in other East Asian populations, including Japan and Thailand, would assess cross-country generalizability and inform the development of a unified East Asian ASCVD risk equation [30]. A dedicated cost-effectiveness analysis, incorporating local treatment costs, side effect profiles, and patient preferences, would determine the optimal decision threshold for the Chinese context. Furthermore, prospective studies of risk equation-guided statin initiation in Chinese primary care are needed to evaluate the real-world clinical impact of these prediction models.

Beyond traditional demographic and metabolic risk factors, emerging evidence suggests that anthropometric phenotyping may capture additional cardiovascular information not represented by established ASCVD risk equations. In particular, noninvasive assessment of chest shape conformation using the modified Haller index (MHI), derived from the ratio of transverse thoracic diameter to the distance between the sternum and spine, has been investigated as a potential prognostic marker. In a cohort of 1,091 patients evaluated for suspected coronary artery disease with exercise stress echocardiography, a concave-shaped chest wall (MHI > 2.5) was independently associated with a significantly lower probability of cardiovascular events over a medium-term follow-up (adjusted hazard ratio (HR), 0.39; 95% CI, 0.26–0.56; P < 0.0001) [31]. While these findings should not be interpreted as establishing chest morphology as a validated ASCVD risk factor, they suggest that simple, noninvasive anthropometric measures may capture incremental cardiovascular information beyond conventional risk factors. Future studies should prospectively evaluate whether adding thoracic or body morphometric variables improves calibration, discrimination, reclassification, or decision-curve net benefit beyond established population-specific risk equations such as K-CVD.

### Conclusions

In this 9-year head-to-head comparison of three country-specific ASCVD risk equations in 9,532 Chinese adults from CHARLS, K-CVD demonstrated the best baseline calibration (predicted/observed = 0.97), while PCE systematically overestimated risk by 17% and China-PAR underestimated risk by 10%. All three equations showed similar discrimination (AUC 0.775–0.777), and the stacking ensemble ML model did not significantly improve discrimination in this low-event-rate cohort. After logit-scale recalibration, all three equations achieved Brier = 0.0116, with slopes of 0.57–0.59, indicating that comprehensive recalibration is required before clinical implementation. DCA revealed that lower decision thresholds (1–2%) may be more appropriate than conventional US/Korean thresholds (7.5–10%) in low-risk Chinese populations.

Three key implications emerge from this study. Among the three country-specific equations tested, K-CVD showed the best baseline calibration in this 9-year Chinese cohort, supporting its consideration as a candidate for East Asian risk harmonization. PCE and China-PAR showed substantial miscalibration in opposite directions (overestimation and underestimation, respectively), highlighting the need for systematic recalibration before clinical use in Chinese populations. DCA suggested that lower thresholds (1–2%) may be more appropriate than conventional 7.5–10% thresholds in low-risk Chinese populations, but cost-effectiveness studies are needed to determine the optimal threshold for the Chinese healthcare context. These findings contribute to the growing body of evidence supporting the development of East Asian-specific ASCVD risk equations and provide practical guidance for ASCVD risk assessment in Chinese primary care.

## Data Availability

The CHARLS data are publicly available from the CHARLS website (https://charls.charlsdata.com) upon registration. The stacking ML code, the recalibration coefficients, and the analysis pipeline are available from the corresponding author upon reasonable request.

## References

[1] Zhou M, Wang H, Zeng X, Yin P, Zhu J, Chen W, Li X (2019). Mortality, morbidity, and risk factors in China and its provinces, 1990-2017: a systematic analysis for the Global Burden of Disease Study 2017. Lancet.

[2] Goff DC, Lloyd-Jones DM, Bennett G, Coady S, D'Agostino RB, Gibbons R, Greenland P (2014). 2013 ACC/AHA guideline on the assessment of cardiovascular risk: a report of the American College of Cardiology/American Heart Association Task Force on Practice Guidelines. Circulation.

[3] Yang X, Li J, Hu D, Chen J, Li Y, Huang J, Liu X (2016). Predicting the 10-year risks of atherosclerotic cardiovascular disease in Chinese population: the China-PAR Project (Prediction for ASCVD Risk in China). Circulation.

[4] Bae JW, Woo SI, Lee J (2023). Development and validation of the korean coronary heart disease risk score (K-CVD). J Lipid Atheroscler.

[5] Li H, Chen S, Zhang R, Tian X, Wu S, Wang A (2026). Validation and recalibration of PCE, China-PAR, and PREVENT models for estimating ASCVD risk in China. JACC Asia.

[6] Yadlowsky S, Hayward RA, Sussman JB, McClelland RL, Min YI, Basu S (2018). Clinical implications of revised pooled cohort equations for estimating atherosclerotic cardiovascular disease risk. Ann Intern Med.

[7] Muntner P, Colantonio LD, Cushman M, Goff DC, Howard G, Howard VJ, Kissela B (2014). Validation of the atherosclerotic cardiovascular disease Pooled Cohort risk equations. JAMA.

[8] Vickers AJ, Elkin EB (2006). Decision curve analysis: a novel method for evaluating prediction models. Med Decis Making.

[9] Vickers AJ, Van Calster B, Steyerberg EW (2016). Net benefit approaches to the evaluation of prediction models, molecular markers, and diagnostic tests. BMJ.

[10] Rhee EJ (2024). Changing pattern of cardiovascular disease in Korea. J Lipid Atheroscler.

[11] Lv J, Yu C, Guo Y, Bian Z, Yang L, Chen Y, Tang X (2017). Adherence to healthy lifestyle and cardiovascular diseases in the Chinese population. J Am Coll Cardiol.

[12] Zhao Y, Hu Y, Smith JP, Strauss J, Yang G (2014). Cohort profile: the China Health and Retirement Longitudinal Study (CHARLS). Int J Epidemiol.

[13] Coresh J, Selvin E, Stevens LA, Manzi J, Kusek JW, Eggers P, Van Lente F (2007). Prevalence of chronic kidney disease in the United States. JAMA.

[14] Jaeger BC, Clark AL, Schaefer AK (2024). Improving the accuracy of pooled cohort equations using cystatin C. J Am Coll Cardiol.

[15] Shlipak MG, Matsushita K, Arnlov J, Inker LA, Katz R, Polkinghorne KR, Rothenbacher D (2013). Cystatin C versus creatinine in determining risk based on kidney function. N Engl J Med.

[16] Levey AS, Stevens LA, Schmid CH, Zhang YL, Castro AF, Feldman HI, Kusek JW (2009). A new equation to estimate glomerular filtration rate. Ann Intern Med.

[17] Steyerberg EW, Vickers AJ, Cook NR, Gerds T, Gonen M, Obuchowski N, Pencina MJ (2010). Assessing the performance of prediction models: a framework for traditional and novel measures. Epidemiology.

[18] DeLong ER, DeLong DM, Clarke-Pearson DL (1988). Comparing the areas under two or more correlated receiver operating characteristic curves: a nonparametric approach. Biometrics.

[19] Pencina MJ, D'Agostino RB, D'Agostino RB, Vasan RS (2008). Evaluating the added predictive ability of a new marker: from area under the ROC curve to reclassification and beyond. Stat Med.

[20] Li C, Liu X, Shen P, Sun Y, Zhou T, Chen W, Chen Q (2024). Improving cardiovascular risk prediction through machine learning modelling of irregularly repeated electronic health records. Eur Heart J Digit Health.

[21] Liu T, Kuo PL, O'Connor S (2025). Machine learning based prediction models for cardiovascular disease risk using electronic health records data: systematic review and meta-analysis. Eur Heart J Digit Health.

[22] Mach F, Baigent C, Catapano AL, Koskinas KC, Casula M, Badimon L, Chapman MJ (2020). 2019 ESC/EAS Guidelines for the management of dyslipidaemias: lipid modification to reduce cardiovascular risk. Eur Heart J.

[23] Arnett DK, Blumenthal RS, Albert MA, Buroker AB, Goldberger ZD, Hahn EJ, Himmelfarb CD (2019). 2019 ACC/AHA guideline on the primary prevention of cardiovascular disease: a report of the American College of Cardiology/American Heart Association Task Force on Clinical Practice Guidelines. Circulation.

[24] Jee SH, Batty GD, Jang Y (2014). The Korean heart study: a prospective cohort study of cardiovascular disease. BMC Cardiovasc Disord.

[25] Lloyd-Jones DM, Braun LT, Ndumele CE, Smith SC, Sperling LS, Virani SS, Blumenthal RS (2019). Use of risk assessment tools to guide decision-making in the primary prevention of atherosclerotic cardiovascular disease: a special report from the American Heart Association and American College of Cardiology. Circulation.

[26] Grundy SM, Stone NJ, Bailey AL, Beam C, Birtcher KK, Blumenthal RS, Braun LT (2019). 2018 AHA/ACC/AACVPR/AAPA/ABC/ACPM/ADA/AGS/APhA/ASPC/NLA/PCNA guideline on the management of blood cholesterol: a report of the American College of Cardiology/American Heart Association Task Force on clinical practice guidelines. Circulation.

[27] Fine JP, Gray RJ (1999). A proportional hazards model for the subdistribution of a competing risk. J Am Stat Assoc.

[28] Chen Z, Chen J, Collins R, Guo Y, Peto R, Wu F, Li L (2011). China Kadoorie Biobank of 0.5 million people: survey methods, baseline characteristics and long-term follow-up. Int J Epidemiol.

[29] Li X, Wu C, Lu J, Chen B, Li Y, Yang Y, Hu S (2020). Cardiovascular risk factors in China: a nationwide population-based cohort study. Lancet Public Health.

[30] Akao H, Polonsky TS, Nakano M (2023). Coronary artery calcium and cardiovascular events in east Asian ethnicities: a systematic review. JACC Asia.

[31] Sonaglioni A, Rigamonti E, Nicolosi GL, Lombardo M (2021). Prognostic value of modified Haller Index in patients with suspected coronary artery disease referred for exercise stress echocardiography. J Cardiovasc Echogr.

